# An Ultrasonic Sensor System Based on a Two-Dimensional State Method for Highway Vehicle Violation Detection Applications

**DOI:** 10.3390/s150409000

**Published:** 2015-04-16

**Authors:** Jun Liu, Jiuqiang Han, Hongqiang Lv, Bing Li

**Affiliations:** 1 School of Electrical Engineering, Xi’an Jiaotong University, Xi’an 710049, China; E-Mail: jliu1912@gmail.com; 2 School of Electronic and Information Engineering, Xi’an Jiaotong University, Xi’an 710049, China; E-Mails: jqhan@xjtu.edu.cn (J.H.); iacxjtu@163.com (B.L.)

**Keywords:** highway vehicle traffic rule violation detection, intelligent transportation systems, two-dimensional state method, ultrasonic sensor system

## Abstract

With the continuing growth of highway construction and vehicle use expansion all over the world, highway vehicle traffic rule violation (TRV) detection has become more and more important so as to avoid traffic accidents and injuries in intelligent transportation systems (ITS) and vehicular *ad hoc* networks (VANETs). Since very few works have contributed to solve the TRV detection problem by moving vehicle measurements and surveillance devices, this paper develops a novel parallel ultrasonic sensor system that can be used to identify the TRV behavior of a host vehicle in real-time. Then a two-dimensional state method is proposed, utilizing the spacial state and time sequential states from the data of two parallel ultrasonic sensors to detect and count the highway vehicle violations. Finally, the theoretical TRV identification probability is analyzed, and actual experiments are conducted on different highway segments with various driving speeds, which indicates that the identification accuracy of the proposed method can reach about 90.97%.

## 1. Introduction

Since the first Bonn-Cologne highway was built in Germany in 1932, there has been continuous highway growth all over the world, in countries such as Germany, the United States, and China. By the end of 2013, the total mileage of China’s highways had reached over 104 thousand kilometers [1], succeeding the United States in having the largest network of highways in the world. At the same time, worldwide vehicle use is also growing very fast, so the highway traffic safety problem has become a significant concern for Intelligent Transportation System (ITS) [2,3]. The driving violations of slower vehicles, especially large heavy trucks, travelling in an improper lane like the “passing lane”, might cause seriously negative effects on the highway traffic order, reduce highway traffic efficiency, and become a safety threat for other drivers who have to change lanes more frequently. It has been statistically found that in China, the total number of road traffic crashes, nonfatal injuries, and fatalities increased by 43-fold, 58-fold and 85-fold, respectively from 1951 to 2008 [4]. Most countries have special traffic laws to avoid accidents on highways, for example, China has announced several important national traffic rules and regulations restricting the improper roadway occupation behavior on highways [5,6], in order to reduce the highway TRV induced accidents.

As there are hundreds of thousands kilometers of highways, it is not easy to monitor all vehicles at any time. Traditional TRV detection methods include: ultrasound based systems [3,7,8,9], capacitive sensor based systems [10], infrared sensors [11], laser and radar sensors [12], traffic video based systems [13], computer vision techniques [2,14], RFID technology [15], *etc.* The former vehicle detection sensors or monitoring cameras were always installed along the highway in fixed positions [16], there might be problems associated with this kind of measurement placement such as: (a) in tunnels or on multiple lane sections of the highways, where sensors might detect vehicles erroneously due to the reception of unnecessary reflected signals; (b) although thousands of sensors have been adopted, they still cannot cover all spots of the highways.

Because the literature has seldom touched the issue of violation detection by moving vehicle measurement or monitoring devices, and most of the past research could not recognize the TRV vehicles in real-time during the whole driving process. This paper proposes a novel ultrasonic sensor system that can perform continuous and reliable TRV detection and counting. The real-time and recorded data from the sensor system can be converted into highway hazard and traffic jam messages, and then be sent to adjacent vehicles (V2V) or roadside infrastructure communication units (V2R, or V2I) through a vehicular *ad hoc* network (VANET) [17,18], so as to improve traffic safety and efficiency. For future implementation of the proposed sensor system, potential challenges will need to be resolved in the fields of efficient medium access control protocol design [19], heterogeneous media provision studies [20], and distributed sensor data fusion algorithms [21], *etc.*, so that safety related and other application messages can be timely and reliably disseminated through vehicular networks.

The structure of the paper is as follows: Section 2 introduces the hardware and software configuration of the ultrasonic sensor system, to be embedded on the host vehicle for highway vehicle TRV detection. The two-dimensional state method to detect the highway vehicle violation is proposed in Section 3, utilizing both the spacial state of the sensors and the past time sequential states being stored. A detailed TRV detection and counting algorithm for the two-dimensional state method is then described, in order to address different driving situations of the vehicles passing-by. Theoretical identification probability for the proposed method is analyzed in Section 4. Real-time experiments on different highway segments with various driving speeds are performed and shown in Section 5, which demonstrates the applicability and high identification accuracy of the proposed method. Finally, the conclusions are given in Section 6.

## 2. Principle of the Ultrasonic Sensor System

After a careful survey and detailed feasibility analysis on different sensor types above, we choose ultrasonic sensors in this study. Ultrasonic sensors have been widely used in ITS and VANET area applications such as vehicle tracking and classification [7,22,23,24,25], obstacle detection and mapmaking [26,27], vacant parking slot detection [28], smart traffic signaling [29], ultrasonic ranging and localization [30], *etc.* Ultrasonic sensors are a well accepted technology for distance sensing applications, because of the inexpensive and easy-to-adopt nature, and reliable and stable measurement performance within their measuring range.

**Figure 1:**
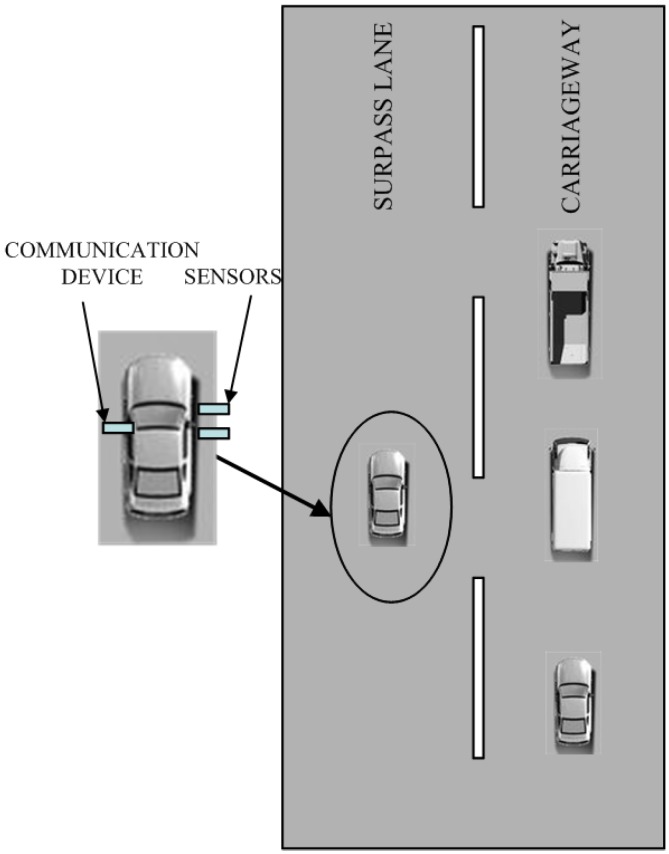
Ultrasonic sensor system for highway vehicle TRV identification.

Figure 1 shows that a vehicle is driving in the passing lane while three other cars are driving on the carriageway. If the speed of the left vehicle is faster but does not exceed the speed limit of that highway, then it will catch up with the three vehicles to its right and surpass them, which is taken as a normal driving behavior; Otherwise, if the speed of the left vehicle is relatively slower, the other three drivers have to pass it on its right side, then this will be considered an illegal TRV behavior of occupying the passing lane (assuming that the country obeys driving on the right). A real-time vehicle tracking system for this TRV driving situation can be designed, by attaching two ultrasonic sensors to the right side of the vehicle, assisted with a communication device for information transfer. While the vehicle is driving on the highway, the two ultrasonic sensors can detect vehicles consistently whether there are other vehicles overtaking it from the lower speed lane to its right side, then the ultrasonic sensor system will record the situation of the vehicle as a TRV behavior.

It might be not reliable to detect a passing vehicle by the movement measurement device with only one ultrasonic sensor, because the reflected signal can be influenced by the target height, length, surface flatness, speed, *etc.* Therefore, the ultrasonic sensor system is designed to contain two parallel ultrasonic sensors, and the measurement data of both sensors can act as a complementary source for each other by using proper data processing techniques, in order to deal with problematic measurements.

**Figure 2:**
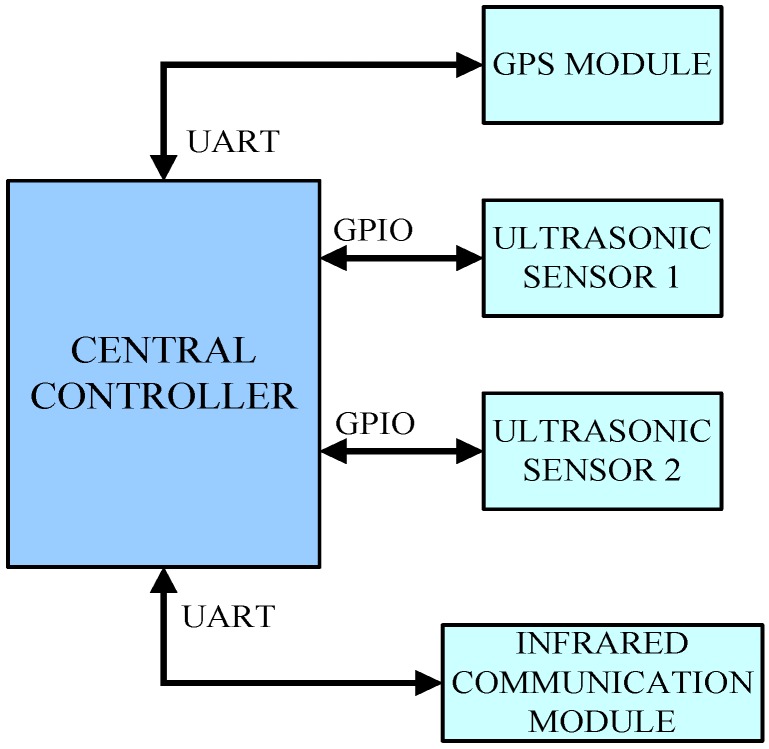
The hardware configuration of the ultrasonic sensor system.

Figure 2 shows the hardware structure of the ultrasonic sensor system, which is comprised of a central controller, a GPS module, an infrared communication module, and two parallel ultrasonic sensors. The ultrasonic sensors send and receive ultrasonic signals to detect whether there are vehicles within their measuring range, and the GPS module provides the current driving speed of the host vehicle. Then the identification algorithms of the central controller will determine whether the driving situation of the host vehicle is an TRV behavior through measurements, and if it is confirmed as an improper lane-overtaking, the violation counter will increase by 1. The infrared communication module will send the results to the receiver in the highway toll station.

**Figure 3:**
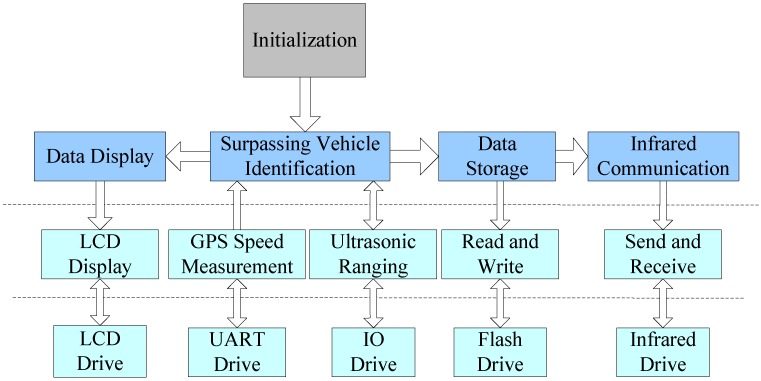
Schematic diagram of the system software functions.

In order to meet the target of recognizing the highway TRV driving behavior, the software of the ultrasonic sensor system should include the following functions: system initialization, passing vehicle identification (including both ultrasonic ranging and GPS speed measurement), data display, data storage, and infrared communication. The schematic diagram of the system software can be shown in Figure 3, and each module contains the corresponding drives and interfaces shown in the figure. The detailed functions can be expressed as follows:

(a) System initialization module, does the following things: running the bootstrap, IO interface configuration, flash configuration, timer initialization, loading the system-related parameters including algorithm related parameters and basic information of the host vehicle (license number, owner information, *etc.*). In addition, the initialization module also completes the variable initialization.
(b) Passing vehicle identification module: identifies whether the vehicle is occupying an improper lane, according to the results measured by the ultrasonic sensors and an on-board GPS. (1) GPS speed measurement module, extracts information of speed, time, latitude and longitude coordinates, according to the frame information from the GPS; (2) Ultrasonic measurement module, controls the ultrasonic sensors to transmit and receive the ultrasound waves, and processes the reflected ultrasonic signal to calculate the distance between vehicles and record the signal strength of each measurement.
(c) Data display module, performs the initialization of the LCD, and displays the TRV recognition and counting results of the host vehicle.
(d) Data storage module, runs the flash initialization, flash read-and-write functions, and then be used to store the configuration parameters, the historical measurement data of ultrasonic sensor and vehicle driving status.
(e) Infrared communication module, provides the infrared communication services for the sensor system to highway toll stations, other vehicles, or roadside communication units.

Among all the modules in the second and third rows of Figure 3, the GPS speed measurement module, ultrasonic ranging module and the surpassing vehicle identification module, are the key subsystems of the software design in the sensor system, thereafter the following sections will introduce the identification method of the three modules in detail.

## 3. The Two-Dimensional State Method for the Ultrasonic Sensor System

As there are real-time measurement data from two ultrasonic sensors, and the host vehicle of the measurement device is moving, the data processing would be quite important so as to deal with all kinds of measurement data.

**Figure 4:**
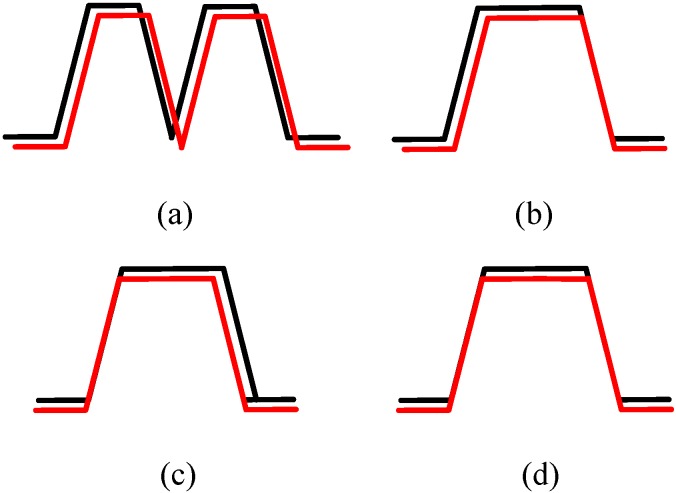
Several examples of typical measurement data of the two parallel ultrasonic sensors. (**a**) Detect the entering and leaving of a passing vehicle continuously; (**b**) Detect the leaving of a passing vehicle concurrently; (**c**) Detect the entering of a passing vehicle concurrently; (**d**) Detect the entering and leaving of a passing vehicle concurrently.

In reality, the detection of a passing or to-be-passed vehicle can be rather complicated, as it might be highly related to the driving habits of the driver, vehicle surface condition, relative speed, relative angle between the target and the host vehicle, *etc.* In order to find the relative movement direction of other vehicles according to measurement data from the two sensors, a feasible way is to record the measurement sequences of both sensors as shown in Figure 4, then discriminate the relative motion direction by the logic analysis of the passing or to-be-passed vehicle. In Figure 4, the black line and red line denote the measurement data from ultrasonic sensor 1 and sensor 2, respectively, the red line data of sensor 2 has been shifted slightly down on the vertical axis for clarity, so as to avoid overlapping of the two colored lines. The identification process is easy to conduct when the data flow is clearly distinguishable (such as the situations of Figure 4b–d), but it might not easily make good judgments when there are breakpoints occurring in the measurement results from one sensor (such as the situation of Figure 4a, when the reflecting surface of the target is not flat, or the measured surface has a relatively large angle with the moving direction of the parallel ultrasonic sensors, then the sensors may not be able to detect the target, and thus there will be breakpoints observed by the sensor system). Therefore, a two-dimensional state method is proposed to address this issue, and to increase the identification rate of the ultrasonic sensor system. The two-dimensional state means the spacial state and the past time sequential states of the two ultrasonic sensors.

### 3.1. Conversion of Binary States of the Ultrasonic Sensors

**Figure 5:**
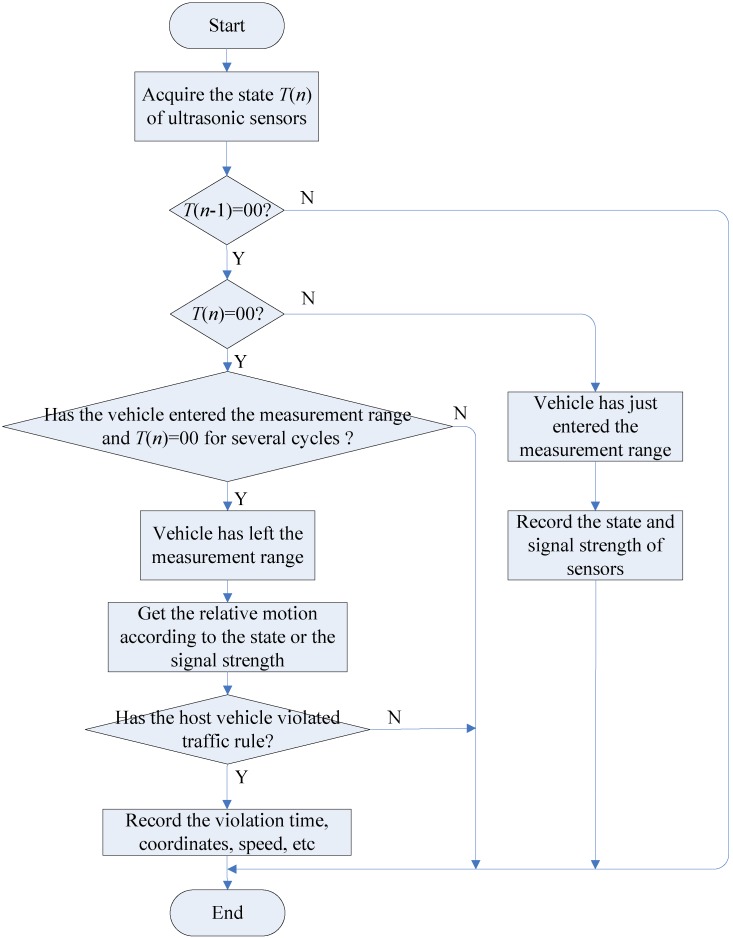
Flowchart of the TRV detection and counting algorithm.

The whole data processing process is shown in Figure 5, which fulfills the main function of traffic-rule-violation detection and counting. In the first step of Figure 5, the controller needs to obtain the state T(n) of the ultrasonic sensors. In fact, the direct measurement data is the one-way travelling distance of the ultrasound wave, which is required to be converted into the binary state of T(n). The binary state *X* of each sensor can be acquired according to the logic rule in Equation (1), which can be 0 or 1, where 0 means that no reflecting signal is received, and 1 means a target is in its measurement scope:

(1) $$X=(d_{meas}>d_{\min})\&(d_{meas}<d_{\max})$$

where: “&” means the logic “and”, $d_{meas}$ is the distance measured by the sensor during this measurement cycle, $d_{\min}$ is the intrinsic blind-area distance of ultrasonic sensors, and $d_{\max}$ is the maximum measured distance or the required effective measurement scope for the ultrasonic sensor system. Then the two sensors’ measuring state can be represented by Equation (2):

(2) $$T\left(n\right)=\left(\left(d_{1}>d_{\min})\&(d_{1}<d_{\max}\right)\right)|\left(\left(d_{2}>d_{\min})\&(d_{2}<d_{\max}\right)\right)$$

where: “|” denotes the separation between the binary states of the two sensors, $d_{1}$ and $d_{1}$ are the measured distances for sensor 1 and sensor 2, respectively.

As it can be seen in Figure 5, after acquiring the ultrasonic sensor state data T(n), the program should determine whether T(n−1) is equal to 00, if T(n−1)≠00, it means the measurement is an intermediate state, and the program is interrupted to load next inputs; Otherwise, continue to inspect whether T(n) is equal to 00, if T(n)=00, then check whether the vehicle has left, and determine the relative motion to the host vehicle, and update the TRV counting number. If T(n)≠00, it suggests that the vehicle has just entered the measuring range, then record the measured states and the reflection signal strength of both ultrasonic sensors. The process of Figure 5 denotes only one measurement cycle of the sensor system, it will continue to run when the data of the next measurement cycle are obtained. During the driving process of the host vehicle on the highway, the sensor system runs the vehicle TRV detection and counting algorithm continuously, it records the TRV numbers, and sends any illegal driving behavior of the host vehicle to the highway toll station.

### 3.2. Spacial State

In the two-dimensional state method, the first dimension is the spacial state *T*(*n*), according to the measured data of the two parallel ultrasonic sensors. The term *T*(*n*) in Equation (2) can be simplified as the expression in Equation (3), where the higher digit $X_{1}$ denotes the sensor measurement result of ultrasonic sensor 1, and the lower digit $X_{2}$ shows the binary state of sensor 2:

(3) $$T(n)=X_{1}X_{2}$$

where *n* denotes the state in time moment *n*, for example the present measurement moment.

If T(n)=01, it means that sensor 2 detects a vehicle in the current moment, and sensor 1 has not detected any vehicle yet. Through this spacial state of the two sensors, this can be used to determine the relative movement direction of a nearby vehicle to the right side. Different values of the lower and higher digits of *T*(*n*) directly indicate the relative movement direction. For example, T(n)=01 means that the vehicle approaches sensor 2 earlier, the relative moving direction is from 2 to 1, the vehicle is trying to overtake the host vehicle, hence the TRV behavior is detected, and the violation counter of the host vehicle increases by 1; Otherwise, if T(n)=10, it means that the vehicle reaches sensor 1 first, so the direction for the binary value of 10 indicates the direction is from 1 to 2, the host vehicle is passing other vehicles, hence the violation counter remains unchanged. It is worth noting that, if it is able to identify the direction of relative movement when the vehicle is entering the sensor measuring range, it will not need to count when the vehicle is leaving the ultrasonic range.

### 3.3. Time Sequential State

If the measurement data is clear, the spacial state is sufficient to distinguish the relative motion direction. However, when the measurement has breakpoints, the spacial state parameter only will not be able to point out correctly the passing lane occupation violations. Therefore, another dimensional state should be considered, the complementary time sequences of *T*(*n* − 1) and *T*(*n* − 2), together with the present measurement *T*(*n*).

If there are other vehicles within the scope of the sensor, but the reflecting surface or the high relative speed of the vehicle, makes the ultrasonic sensors miss the ultrasound reflection occasionally, the measurement sequence will display a certain number of breakpoints. In this kind of situation, the primary concern should be determining whether the vehicle has left completely, which can avoid double TRV counting of the sensor system. Since all vehicles are required to maintain a certain safety distance to the vehicle ahead on the highway, the TRV counter will increase only when the moving object has left for at least two measurement cycles. Otherwise, the counter remains unchanged. The situation with breakpoints can be divided into several conditions, according to the previous state values of *T*(*n* − 1) at the time moment of (*n* − 1).

#### 3.3.1. *T*(*n* − 1) = 00

**Figure 6:**
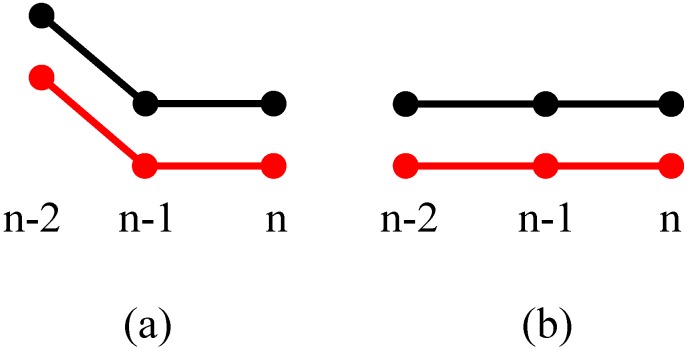
*T*(*n* − 1) = 00 and *T*(*n*) = 00. (**a**) An vehicle has been detected to be in the measuring range of the sensor system before; (**b**) No vehicle has been detected in the sensor's measuring scope before.

For the situation when the present state is *T*(*n*) = 00, and an object has been detected to be in the measuring range of the sensor system before, then it must decide whether the vehicle has completely driven out of the sensor measurement range. If the vehicle has left, the relative movement direction of the vehicle is from 2 to 1, and the violation counter increases by 1. If *T*(*n*) = 00 and no vehicle has been detected in the sensor’s measuring scope before, then the counter does not count. These two conditions can be simply shown in Figure 6a,b with binary state plots.

**Figure 7:**
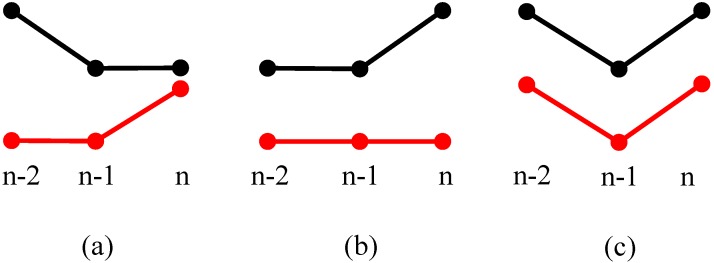
*T*(*n* − 1) = 00 and *T*(*n*) ≠ 00. (**a**) *T*(*n*) = 01; (**b**) *T*(*n*) = 10; (**c**) *T*(*n*) = 11.

If the present state is T(n)≠00, Figure 7 shows three different situations of (a) T(n)=01, (b) T(n)=10 and (c) T(n)=11. Figure 7a indicates that sensor 1 (black dotted line) might have two continuous breakpoints and sensor 2 (red dotted line) might have missed one reflecting signal at moment *n*. Figure 7c means that both two sensors experience a breakpoint at moment *n*. Figure 7b indicates there might be another vehicle that is entering the ultrasonic sensor measurement range of sensor 1, or there might be at least two continuous breakpoints for both ultrasonic sensors at (n−2) and (n−1), it will depend on more measurement results to give a correct identification in this situation.

#### 3.3.2. *T*(*n* − 1) ≠ 00

**Figure 8:**
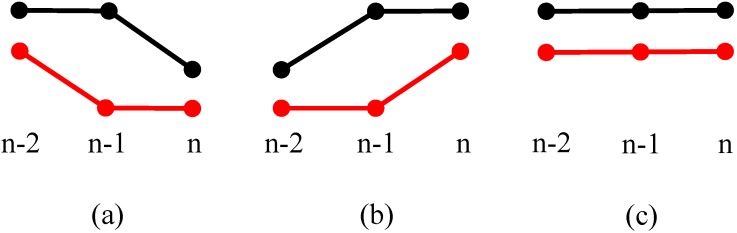
*T*(*n* − 1) ≠ 00. (**a**) A vehicle has just left the measuring range; (**b**) A vehicle enters the measuring range of sensor 1 and 2 continuously; (**c**) A vehicle is passing through both sensors’ measurement range.

For the situation when the present state as *T*(*n* − 1) ≠ 00, three different situations are shown in Figure 8. Figure 8a can be a vehicle that has just left the measuring range, so both sensors display a state with no vehicles for the first time, and it needs to wait for the following measurement cycle so as to avoid the breakpoint judgment. Figure 8b indicates a situation where a new rising edge is being detected by both sensors, the vehicle enters the measuring range of sensor 1 first, and then enters the measuring range of sensor 2. Figure 8c shows three continuous positive values for both sensors, which denotes a moving target is passing through the sensors’ coverage, and both sensors detect the moving target at (n−2), (n−1) and *n* time moments.

### 3.4. Improvement by Measured Reflection Signal Strength

**Figure 9:**
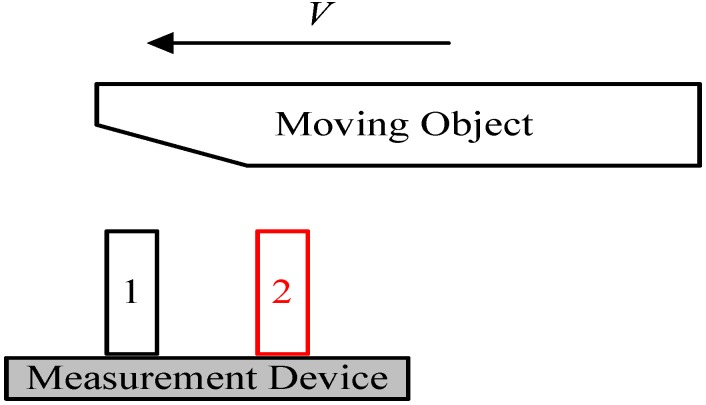
Diagram for a moving object with varying reflecting surfaces.

Typically, the binary spacial and past time sequential states are sufficient to successfully identify the TRV behavior. However, if the vehicle remains in the measuring range of both ultrasonic sensors simultaneously, such as the situation described in Figure 4d and Figure 8c, the binary states will be 11 for T(n−2), T(n−1) and T(n), thus it would be unable to determine the direction of movement of the object. Under this circumstance, decisions will be made according to the measured reflection signal strength. The principle is that, the head and tail part of the moving vehicle is always not flat enough, or has some intersection angle with the ultrasonic sensor plane, as shown in Figure 9, hence the ultrasonic reflection signals by these surfaces are usually weak. For example, although sensor 1 and 2 both detected the moving object at the same moment in Figure 9, the reflection signal of sensor 2 is much bigger than that of sensor 1; therefore, we can set a proper threshold value Δd for the signal strength difference between the two sensors, if the signal strength of sensor 2 minus that of sensor 1 is greater than the threshold Δd, it can be seen that the motion direction is from 2 to 1. Similarly, when a moving object is leaving the measuring range, the signal strength difference will also be applicable.

## 4. Theoretical Identification Accuracy Analysis

In the real world, the traffic conditions can be very complicated, the host vehicle might overtake other cars, and other cars might overtake the host vehicle. A simple probability analysis on the TRV identification accuracy of the ultrasonic sensor system is performed, according to the random driving situation of the measurement device and the passing vehicle.

### 4.1. Conditions for Successful Identification of TRV Behavior

In order to estimate the probability of the TRV identification algorithm, the condition for successful identification of TRV behavior should be analyzed first. Assuming that the distance between the two ultrasonic sensors is d, the ultrasonic sending direction is perpendicular to the driving direction of the host vehicle with a time interval of T. The relative speed of a passing or to-be-passed vehicle with respect to the host vehicle is assumed to be a constant speed V, because the passing time of other vehicles through the measuring unit is typically very short in our tests, with a minimum of less than 1 s, and no more than 10 s for the longest situation, so this constant relative speed assumption is reasonable.

#### 4.1.1. When Other Vehicles Start to Enter the Measurement Range

**Figure 10:**
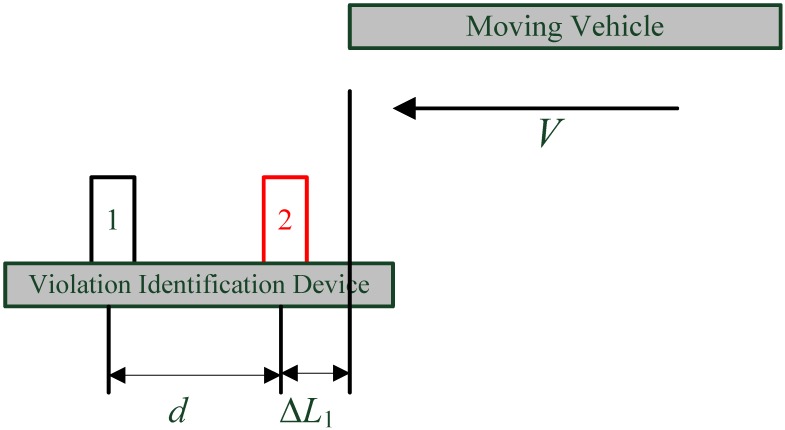
A vehicle is about to enter the measurement range of the sensor system.

As it can be seen in Figure 10, another vehicle is approaching the measuring device a measurement cycle *T* before, the present distance of the head of the vehicle to sensor 2 is $\Delta L_{1}$, and $0<\Delta L_{1}<VT$.

**Figure 11:**
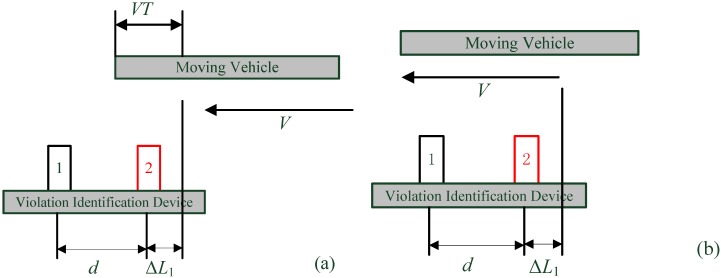
Vehicle has just entered the measurement range: (**a**) Detected by sensor 2 only; (**b**) Detected by both sensors.

An ultrasonic measurement cycle *T* later, the relative travel between the vehicle and the measuring device will be VT, then the relative position of vehicle and the sensor system can be as seen in either Figure 11a or Figure 11b. Figure 11a shows that only ultrasonic sensor 2 detects the passing vehicle, and ultrasonic sensor 1 has not sensed any object at this moment, so the relative motion direction of the vehicle can be easily identified as from 2 to 1. The other vehicle is catching up to the host vehicle, which indicates that the host vehicle is driving in an improper lane; Figure 11b indicates that both ultrasonic sensors detect the passing vehicle, which can also been identified as a TRV of roadway overtaking behavior as Figure 11a does. It can be found in Figure 11 that the inequality relationship of Equation (4) must be satisfied, so as to successfully identify the relative motion direction of the passing vehicle:

(4) $$d+\Delta L_{1}>VT$$

#### 4.1.2. When Other Vehicles Start to Leave the Measurement Range

**Figure 12:**
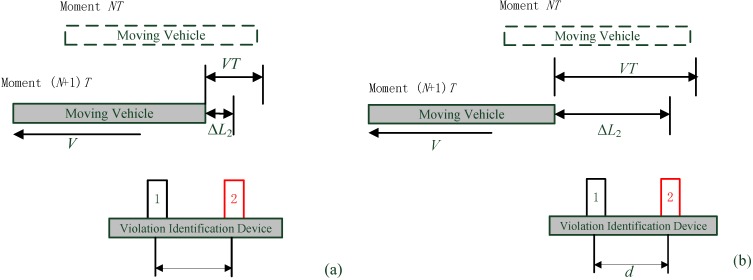
Vehicle is leaving the measurement range: (**a)** Detected by sensor 1 only; **(b)** Detected by no sensor.

Figure 12 shows that another vehicle is leaving the ultrasonic sensors’ measuring range, and both ultrasonic sensors can detect the vehicle a measurement cycle before, but at this moment ultrasonic sensor 1 is able to receive its reflecting signal and sensor 2 fails as indicated in Figure 12a, while neither ultrasonic sensor can detect the vehicle in the situation of Figure 12b.

According to Figure 12a, the inequality relationship of Equation (5) must be satisfied, so as to identify the relative motion direction of the leaving vehicle:

(5) $$0<\Delta L_{2}<d$$

It should be noted that $\Delta L_{2}<VT$ must be true in this situation, because both ultrasonic sensors can detect the vehicle a cycle before the state of Figure 12a. The distance $\Delta L_{2}$ is the distance from the tail of the vehicle to sensor 2, which is obviously related to both the distance $\Delta L_{1}$ and the vehicle length *L*. Since Equations (4) and (5) can both be satisfied if d>VT, only the d<VT situation should be analyzed further so as to accomplish a successful TRV identification.

From the driving state of Figure 10 to Figure 12a, it must have experienced an integer number of measurement cycles, assuming that there have been *N* cycles. During this period, the surpassing vehicle has gone through a travel of $L+\Delta L_{1}+\Delta L_{2}$, corresponding to the position of sensor 2:

(6) $$L+\Delta L_{1}+\Delta L_{2}=NVT$$

where $0<\Delta L_{1}$, and $\Delta L_{2}<VT$.

Assuming that $L=kVT+\Delta L_{3}$, the new variable $\Delta L_{3}$ being introduced, is related only to the vehicle length, and it must satisfy $0<\Delta L_{3}<VT$, and k<N, so substituting these relationships into Equation (6) yields:

(7) $$\Delta L_{1}+\Delta L_{2}+\Delta L_{3}=(N-k)VT$$

where $\Delta L_{1}$ relates to the time when the vehicle enters the measurement range, and $\Delta L_{3}$ relates to the vehicle length, therefore, the two variables are independent of each other, and can be seen as statistically independent random variables.

Because $0<\Delta L_{1}<VT$ and $0<\Delta L_{3}<VT$, then $0<\Delta L_{1}+\Delta L_{3}<2VT$. Thus Equation (7) can be classified into two cases, firstly, $0<\Delta L_{1}+\Delta L_{3}<VT$, and (N−k) can only be equal to 1 in this case, otherwise $\Delta L_{2}$ will be larger than *VT*, therefore, we have:

(8) $$\Delta L_{1}+\Delta L_{2}+\Delta L_{3}=VT,\;\;\text{if}\;\;\;0<\Delta L_{1}+\Delta L_{3}<VT$$

Secondly, when $VT<\Delta L_{1}+\Delta L_{3}<2VT$, and (N−k) should be equal to 2 in this case, otherwise $\Delta L_{2}$ will be larger than *VT*, then we have:

(9) $$\Delta L_{1}+\Delta L_{2}+\Delta L_{3}=2VT,\;\;\text{if}\;\;VT<\Delta L_{1}+\Delta L_{3}<2VT$$

Combining Equations (5), (8) and (9), the following inequality relationships must be satisfied in order to identify the relative motion direction of the leaving vehicle:

(10) $$VT-\Delta L_{1}-\Delta L_{3}<d,\;\;\text{if}\;\;\;0<\Delta L_{1}+\Delta L_{3}<VT$$

or:

(11) $$2VT-\Delta L_{1}-\Delta L_{3}<d,\;\;\text{if}\;\;\;VT<\Delta L_{1}+\Delta L_{3}<2VT$$

According to the analysis above, the parameters should meet the condition of Equation (4) plus Equations (10) or (11) so as to successfully identify a passing or to-be-passed vehicle with the sensor system.

### 4.2. Theoretical Identification Probability for the Proposed Method

Equations (4), (10) and (11) are all related to parameter $\Delta L_{1}$, which is an random variable. According to the basic knowledge of probability theory, the summation of probability of identified and unidentified situations for the proposed method should be 1. That is:

(12) $$P_{identified}=1-P_{unidentified}$$

Based on the information from Equations (4), (10) and (11), the condition that fails to identify the direction of the vehicle movement, should be the dissatisfaction of “Equation (10) plus Equation (4)” or “Equation (11) plus Equation (4)”, which can be expressed as:

(13) $$\{\begin{array}{c} VT-\Delta L_{1}-\Delta L_{3}>d\\ 0<\Delta L_{1}+\Delta L_{3}<VT\\ \Delta L_{1}+d<VT \end{array}$$

or:

(14) $$\{\begin{array}{c} 2VT-\Delta L_{1}-\Delta L_{3}>d\\ VT<\Delta L_{1}+\Delta L_{3}<2VT\\ \Delta L_{1}+d<VT \end{array}$$

In Equation (13), the first expression $VT-\Delta L_{1}-\Delta L_{3}>d$ can be rewritten as $\Delta L_{1}+\Delta L_{3}<VT-d$, together with the other two expressions, $0<\Delta L_{1}+\Delta L_{3}<VT$ and $\Delta L_{1}+d<VT$, Equation (13) can be simplified as Equation (15):

(15) $$\Delta L_{1}+\Delta L_{3}<VT-d$$

Similarly in Equation (14), the first expression can be rewritten as $\Delta L_{1}+\Delta L_{3}<2VT-d$, which has already been included in the second expression of $\Delta L_{1}+\Delta L_{3}<2VT$, then Equation (14) can be simplified as Equation (16):

(16) $$\{\begin{array}{c} VT<\Delta L_{1}+\Delta L_{3}<2VT-d\\ \Delta L_{1}+d<VT \end{array}$$

Therefore, the probability of identified situations for the proposed method will be:

(17) $$P_{identified}=1-P\left\{\Delta L_{1}+\Delta L_{3}<VT-d\right\}-P\{VT<\Delta L_{1}+\Delta L_{3}<2VT-d,\;\Delta L_{1}<VT-d\}$$

where $0<\Delta L_{1}<VT,\;\;\Delta L_{3}<VT<d$.

As is known from the analysis above, $\Delta L_{1}$ and $\Delta L_{3}$ are independent of each other, and they can be seen as obeying a uniform distribution along the range of (0, VT). Then we can calculate the two terms of Equation (17) separately as:

(18) $$\begin{array}{l} P\left\{\Delta L_{1}+\Delta L_{3}<VT-d\right\}\\ =\int_{0}^{VT-d}\int_{0}^{VT-d-\Delta L_{1}}\frac{1}{{\left(VT\right)}^{2}}d\left(\Delta L_{1}\right)d\left(\Delta L_{3}\right)=\int_{0}^{VT-d}(\frac{VT-d-\Delta L_{1}}{{\left(VT\right)}^{2}})d\left(\Delta L_{1}\right)\;=\frac{{\left(VT-d\right)}^{2}}{2{\left(VT\right)}^{2}} \end{array}$$

(19) $$\begin{array}{l} P\left\{VT<\Delta L_{1}+\Delta L_{3}<2VT-d,\;\Delta L_{1}<VT-d\right\}\\ \;\;\;\;=\int_{0}^{VT-d}\int_{VT-\Delta L_{1}}^{2VT-d-\Delta L_{1}}\frac{1}{{\left(VT\right)}^{2}}d\left(\Delta L_{1}\right)d\left(\Delta L_{3}\right)=\int_{0}^{VT-d}(\frac{VT-d}{{\left(VT\right)}^{2}})d\left(\Delta L_{1}\right)=\frac{{\left(VT-d\right)}^{2}}{2{\left(VT\right)}^{2}} \end{array}$$

Substituting Equations (18) and (19) into Equation (17), the probability of identified situations can be obtained as:

(20) $$P_{identified}=1-\frac{{\left(VT-d\right)}^{2}}{{\left(VT\right)}^{2}},\;\;\;\text{if}\;d<VT$$

When d>VT, the condition (4) and (5) will surely be satisfied, then the probability of identified situations under the condition of d>VT will be:

(21) $$P_{identified}=1,\;\;\;\text{if}\;d>VT$$

**Figure 13:**
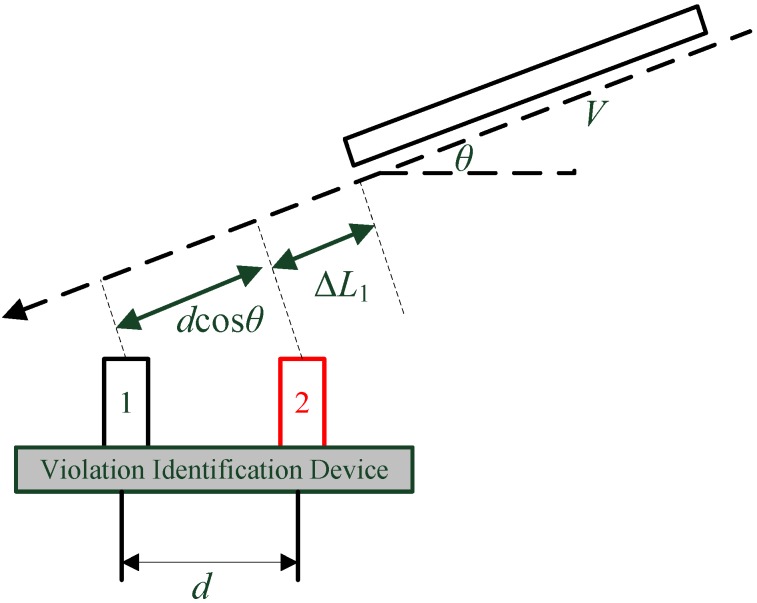
Driving direction of other vehicles is not parallel with the violation identification device.

Equations (20) and (21) give the theoretical probability of the TRV identification algorithm by the proposed ultrasonic sensor system and the two-dimensional state method. In addition, when the driving direction of the passing vehicle has a certain angle θ with the measurement system, such as the situation being shown in Figure 13, only the ultrasonic reflection that is perpendicular to the vehicle surface can be received by the sensor. Therefore, the measurement distance should be corrected by a certain coefficient, namely, replacing the measurement distance d by dcosθ in Equations (20) and (21). The final probability considering the angle between the sensor system and the target vehicle driving direction will be:

(22) $$P_{identified}=\{\begin{array}{ll} 1-\frac{{\left(VT-d\cos\theta \right)}^{2}}{{\left(VT\right)}^{2}}, & d\cos\theta <VT\\ 1, & d\cos\theta \geq VT \end{array}$$

It can be concluded from the analysis above and Equation (22) that: (a) The smaller the relative speed *V*, the bigger the probability *P*. (b) The smaller the measurement time interval *T*, the bigger the probability *P*. (c) The greater the spacing *d* between the two sensors, the bigger the probability *P*. (d) The smaller the angle θ, the greater the probability *P*, and the angle should not be larger than the ultrasound beam angle, otherwise, the ultrasonic sensors will not be able to receive the reflection wave.

Since the length of the host vehicle is limited and the relative speed *V* can vary, the theoretical value $P_{identified}=1$ in the situation of d>VT cannot be easily achieved.

## 5. Experimental Results

**Figure 14:**
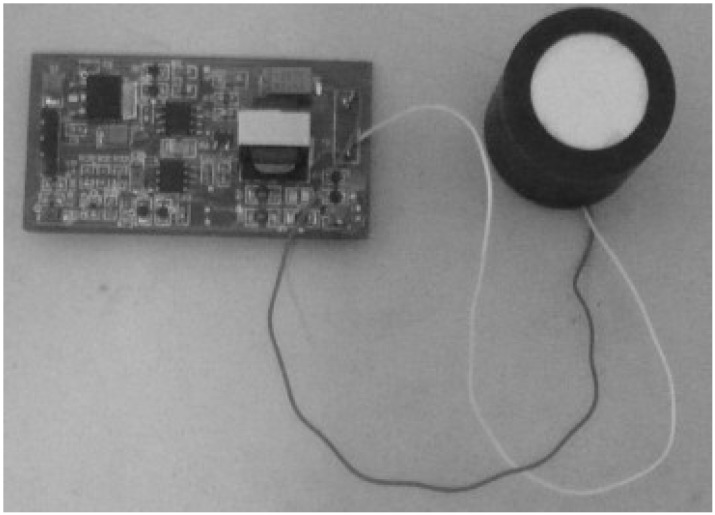
The physical connection of the ultrasonic sensor with the control board for TRV identification experiments.

**Figure 15:**
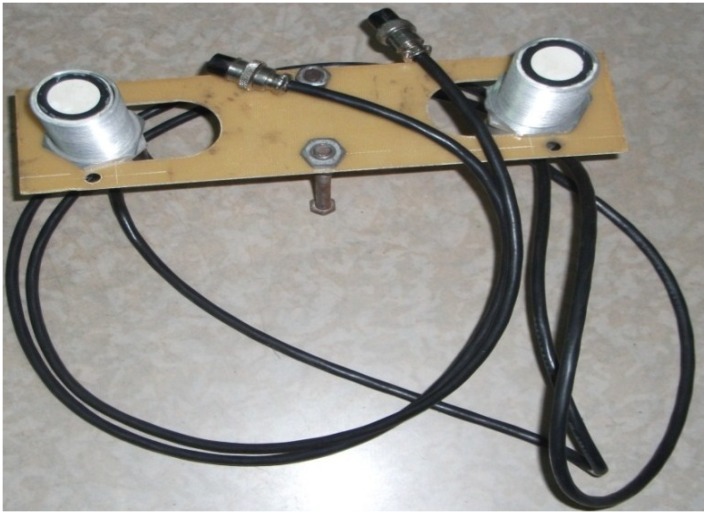
The two parallel ultrasonic sensors fixed on one board.

**Figure 16:**
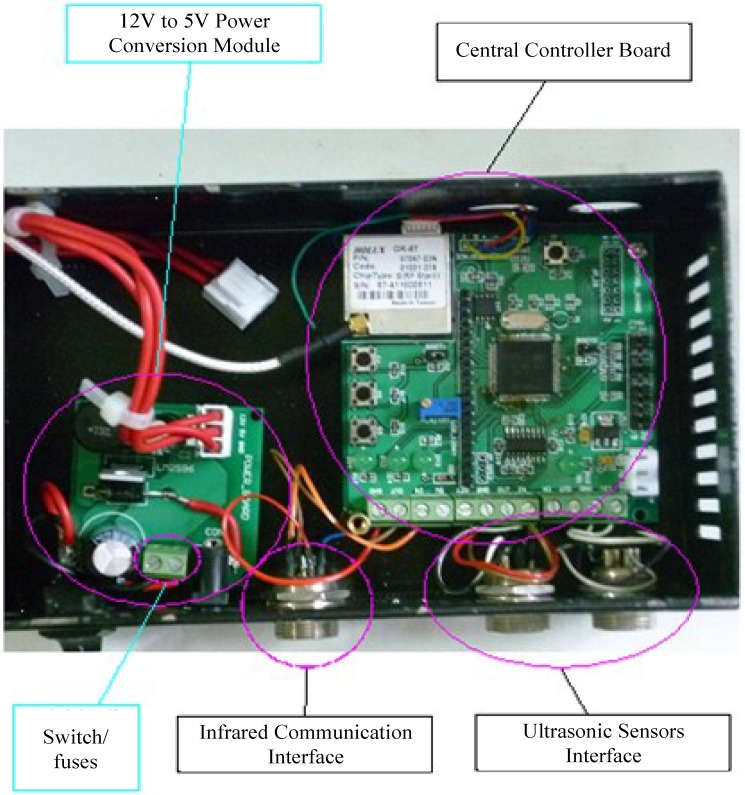
The layout of the central controller board and related interfaces.

According to the hardware and software configuration from Section 2, the physical connection of the ultrasonic sensor with the control board can be seen in Figure 14, the placement of the two sensors is shown in Figure 15, and the developed central controller board and related interfaces for the TRV identification experiments are shown in Figure 16.

In the ultrasonic measuring devices, the corresponding timer accuracy is 5 μs (frequency resolution is chosen as 100 kHz, since the higher the frequency, the stronger the reflection ability; a typical ultrasound wave has a frequency of more than 20 kHz), assuming the ultrasonic speed in air is about constant at 340 m/s, the distance measuring accuracy will be (340 m/s × 5 μs)/2 = 1.7 mm, thus the distance resolution of the ultrasonic sensor is 1.7 mm. The maximum measuring distance of the ultrasonic sensor is chosen as 3.4 m (approximately the length of one lane on the highway) because it is not reasonable to punish the driver when there are more than two vacant lanes to his/her right side. The ultrasonic sensor can perform a distance measurement every 20 ms, considering the additional calculation time by the TRV detection algorithm, so the actual measuring time interval for the ultrasonic sensors is set as 30 ms. All the sensor parameters are listed in Table 1.

**Table 1:** Parameters of the ultrasonic sensors.

Frequency	Minimum Distance	Maximum Distance	Ultrasonic Accuracy	Measurement Interval
100 kHz	0.35 m	3.4 m	1.7 mm	30 ms

**Figure 17:**
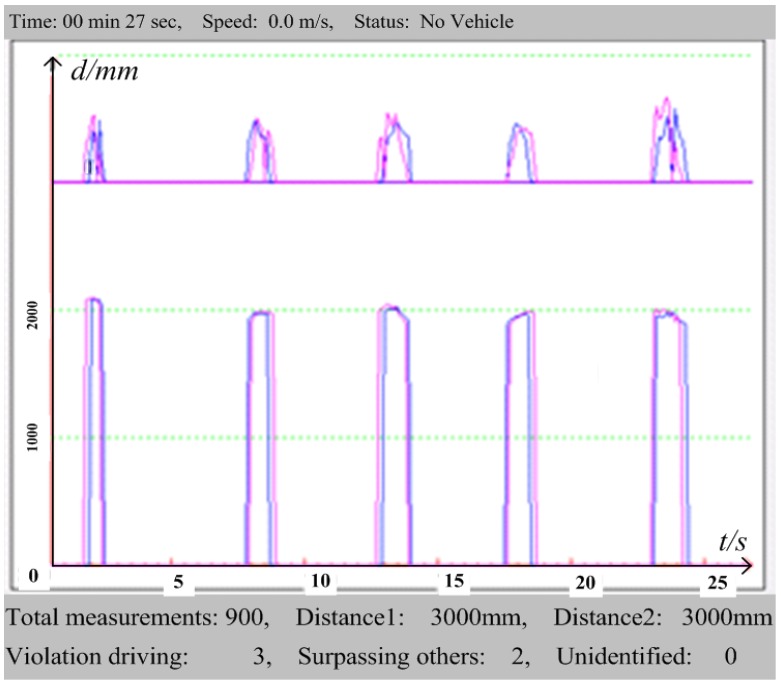
Reflection signal strength and real-time distance measured by the sensors and displayed on PC software.

An experimental ultrasonic distance measurement test has been recorded and transferred via a serial port to the software developed on PC, and the corresponding graphical display is presented in Figure 17.

It can be seen that there are five vehicles being detected by the proposed method within the 27 s test, among which there are three TRV of the host vehicle identified (blue line of sensor 1 lagging the red line of sensor 2), and 2 legal passings of other vehicles (red line lagging the blue line). The upper curve denotes the real-time signal strength measurement data, and the lower curve shows the measured distances of every ultrasonic reflection signal for both sensors. As previously discussed in Section 3, TRV identification can be made according to the binary states converted from the measured distance data from the lower curve, if the binary states are distinguishable. However, the upper signal strength curve must be considered when there are breakpoints detected by the ultrasonic sensors, and this complementary curve can make the identification algorithm more robust.

More experiments on different highway segments with various driving speeds, have been conducted on the G65 highway of China, and the TRV counting numbers of the proposed identification method and the counting results by human observation are compared in Table 2 and Table 3.

It should be noted that Table 2 uses the ultrasonic distance values only, and Table 3 uses the improved method with the assistance of reflection signal strength difference between the two sensors. It can be seen that the accuracy of Table 3 is comparatively higher than Table 2 when the host vehicle has the same driving speed, because the additional information of the signal strength information can improve the identification accuracy. There is an abnormal count in the first case of Table 3, because a long truck with a very low relative speed and unsmooth surface was passing our host vehicle, and the vehicle has been counted twice during one passing action. To sum up, the error rate of the proposed method can reach 9.03% and 2.91% respectively, without and with the additional signal strength data. In addition, because of the high-frequency and good-reflective characteristics of ultrasonic sensors with the specific measurement range of 0.35–3.4 m, weather conditions, such as rainy, partly-cloudy weather or even night applications, have little influence on the sending and receiving of signals during our tests.

**Table 2:** Experimental results *without* signal strength on 7 June 2014.

Road Segments	Driving Speed (km/h)	Tested Mileage (km)	No. of System Counter	No. by Human Observation	Error Rate
Jingyang to Sanyuan	60	14	46	50	8%
Sanyuan to Tongchuan	70	28	45	49	8.2%
Tongchuan to Sanyuan	70	28	32	36	11.1%
Sanyuan to Jingyang	60	14	18	20	10%
Total	-	84	141	155	**9.03%**

**Table 3:** Experimental results *with* signal strength on 13 July 2014.

Road Segments	Driving Speed (km/h)	Tested Mileage (km)	No. of System Counter	No. by Human Observation	Error Rate
Caotan to Jingyang	60	16	65	64	−1.6%
Jingyang to Sanyuan	70	14	38	40	5%
Sanyuan to Tongchuan	70	28	57	58	1.7%
Tongchuan to Jingyang	80~100	42	40	44	9.1%
Total	-	100	200	206	**2.91%**

If the parameters are assumed as constants of *V* = 10 m/s, *T* = 30 ms, *d* = 0.18 m and θ=0°, then the theoretical identification rate will be P=84% according to the probability analysis in Equation (22) of Section 4. The actual measurement results from Table 2 and Table 3 have a slightly higher detection rate of over 90.97%, because the relative driving speeds are not always a constant for different passing situations, if the relative speed of the passing vehicles with respect to the host vehicle are less than 10 m/s, the detection rate will be higher than the theoretical value calculated using Equation (22).

## 6. Conclusions

The paper aims to track the slower vehicles that are occupying the passing lane of highways for a certain time, which might lead to traffic jams or driving safety problems. A novel ultrasonic sensor system to detect this kind of TRV behavior on a moving measurement device is developed, by monitoring the driving status of other passing vehicles in real-time. Accordingly, a two-dimensional state method is proposed to fulfill the function of TRV detection and counting. The sensor system is comprised of two parallel ultrasonic sensors to scan the passing vehicles, and the distances measured by both sensors are converted into the binary spacial states, and the historical stored measurement data act as the time sequential states, to perform more reliable highway TRV behavior detection. Through the monitoring of the changes of the two-dimensional states, the relative motion direction of other vehicles can be recognized, the theoretical identification rate is analyzed according to the random driving situation of the measurement device and the passing vehicles. Experiments have shown that the proposed ultrasonic sensor system is able to identify the improper TRV driving behavior of the host vehicle to an accuracy of about 90.97%. The proposed ultrasonic sensor system, as well as the TRV detection and counting method will be a significant supplement in intelligent transportation systems (ITS) and vehicular *ad hoc* networks (VANETs), so as to avoid traffic accidents and injuries.
